# Graphitic carbon growth on crystalline and amorphous oxide substrates using molecular beam epitaxy

**DOI:** 10.1186/1556-276X-6-565

**Published:** 2011-10-26

**Authors:** Sahng-Kyoon Jerng, Dong Seong Yu, Jae Hong Lee, Christine Kim, Seokhyun Yoon, Seung-Hyun Chun

**Affiliations:** 1 Department of Physics and Graphene Research Institute, Sejong University, Seoul 143-747, South Korea; 2 Department of Physics, Ewha University, Seoul 151-747, South Korea

**Keywords:** graphite, molecular beam epitaxy, Raman, oxide

## Abstract

We report graphitic carbon growth on crystalline and amorphous oxide substrates by using carbon molecular beam epitaxy. The films are characterized by Raman spectroscopy and X-ray photoelectron spectroscopy. The formations of nanocrystalline graphite are observed on silicon dioxide and glass, while mainly *sp^2 ^*amorphous carbons are formed on strontium titanate and yttria-stabilized zirconia. Interestingly, flat carbon layers with high degree of graphitization are formed even on amorphous oxides. Our results provide a progress toward direct graphene growth on oxide materials.

**PACS**: 81.05.uf; 81.15.Hi; 78.30.Ly.

## Introduction

Graphene growth on Ni or Cu by chemical vapor deposition [CVD] is now well established. However, the CVD graphene needs to be transferred onto insulating substrates for application, which may degrade the quality and bring complications to the manufacturing process. This is why direct graphene growth on insulator is still intensively being studied. Notably, the growth on oxide is of great interest because graphene is expected to face current metal-oxide semiconductor [MOS] technology through an oxide layer. Recent studies have shown some accomplishments toward this goal by using CVD [1-3].

Here, we attempt molecular beam epitaxy [MBE] of carbon onto several oxide substrates to figure out the potential of graphene growth. So far, carbon MBE has been applied mostly on group IV semiconductors [4-7], where graphitic carbon growth was observed. We have shown previously that nanocrystalline graphite [NCG] can be formed on sapphire (Al_2_O_3_) and observed a Dirac-like peak for the first time in MBE-grown NCGs [8]. In this study, we expand the subject to include various crystalline and amorphous oxides. We observe that graphitic carbon or NCG can be grown by carbon MBE on amorphous SiO_2_, the most important oxide in the MOS technology. We also obtain similar results on glass (Eagle 2000™, Corning Inc., Corning, NY, USA). In contrast, carbons on amorphous TiO_2 _or Ta_2_O_5 _do not seem to form graphitic structures. Among the crystalline oxides, mainly *sp^2 ^*amorphous carbons are observed on SrTiO_3_(100) and yttria-stabilized zirconia [YSZ] (100).

## Methods

### Materials and film fabrication

Samples were fabricated in a home-made ultra-high-vacuum MBE system. Carbons were sublimated from a heated pyrolytic graphite filament. The pressure of the chamber was kept below 1.0 × 10^−7 ^Torr during the growth with the help of liquid nitrogen flowing in the shroud. Details about the growth procedure can be found elsewhere [8]. Both crystalline and amorphous oxide substrates were purchased from commercial vendors (AMS Korea, Inc., Sungnam, Gyeonggi-do, South Korea; INOSTEK Inc., Ansan-si, Gyeonggi-do, South Korea). The growth temperature (*T*_G_) was in the range of 900°C to approximately 1,000°C, based on our previous study with sapphire. The typical thickness of carbon film, determined by measuring the step height after lithography, was 3 to approximately 5 nm.

### Characterization

Raman-scattering measurements were performed by using a McPherson model 207 monochromator with a 488-nm (2.54 eV) laser excitation source. The spectra recorded with a nitrogen-cooled charge-coupled device array detector. X-ray photoelectron spectroscopy [XPS] measurements to analyze carbon bonding characteristics were done by using a Kratos X-ray photoelectron spectrometer with Mg Kα X-ray source. C1s spectra were acquired at 150 W X-ray power with a pass energy of 20 eV and a resolution step of 0.1 eV. Atomic force microscopy [AFM] images were taken by a commercial system (NanoFocus Inc., Seoul, South Korea) in a non-contact mode.

## Results and discussion

Raman-scattering measurements have become a powerful, non-destructive tool in the study of *sp^2 ^*carbons (carbon nanotube, graphene, and graphite). The well-known *G *peak is observed in all *sp^2 ^*systems near 1,600 cm^-1^. With the advent of graphene, the so-called *2D *peak, which occurs near 2,700 cm^-1^, has become important. Single-layer graphene is characterized by the sharp and large *2D *peak. This *2D *peak is actually the second order of *D *peak. The typical position of *D *peak is 1,350 cm^−1^, one half of the *2D *peak position. The *D *peak is absent in a perfect graphene sheet or graphite because of symmetry and increases as defects or disorders in the honeycomb structure increases. However, it should be noted that the *D *peak also disappears in amorphous carbon. That is, Raman *D *peak does indicate the presence of sixfold aromatic rings as well as *sp^2 ^*bonds. It is from A_1g _symmetry phonons in which the *D *peak becomes Raman active by structural disorders in the graphene structure.

**Figure 1:**
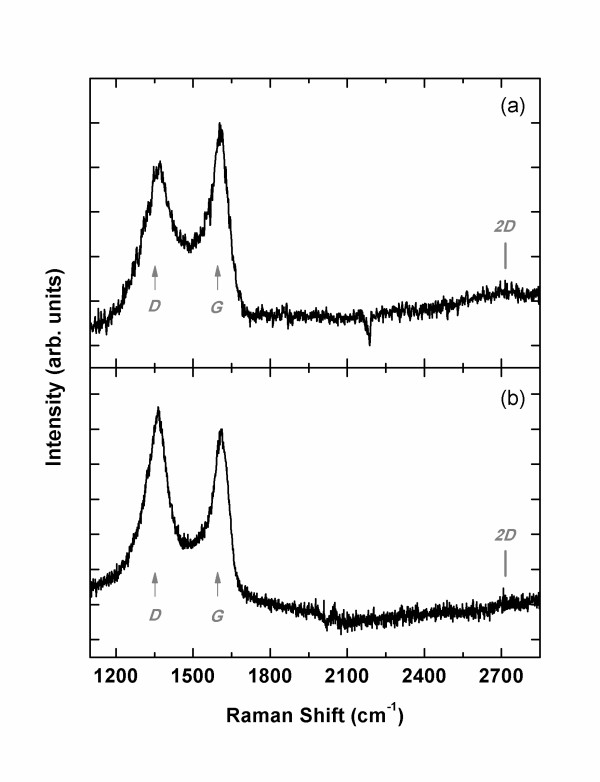
**Raman spectra of carbon films**. The films were grown (**a**) at 1,000°C on SrTiO_3_(100) and (**b**) at 900°C on YSZ(100). The *D *and the *G *peaks are identified.

Ferrari and Robertson studied the degree of *sp^2 ^*bonding and the relative strength of *D *and *G *peaks thoroughly [9-11], and recent experiments confirmed their theory [12,13]. Here, we follow their arguments and evaluate the degree of crystallinity based on the sharpness and the intensity of *D*, *G*, and *2D *peaks. Let us start with carbon deposited on crystalline oxide substrates. Figure 1 shows the Raman spectra from the carbon films grown on SrTiO_3_(100) and YSZ(100). The well-developed *D *and *G *peaks with similar intensities indicate that the film consists of *sp^2 ^*carbons with a number of defects. However, the *2D *peak is hardly seen although a small bump is observed at the expected position in Figure 1a. According to recent criteria, the absence of a clear *2D *peak implies the transition from NCG to mainly *sp^2 ^*amorphous carbon [11]. Based on the intensity ratio, *I_D_*/*I_G _*~ 1 (Table 1), we can conclude that the carbon films on SrTiO_3_(100) and YSZ(100) are in the middle of 'stage 2' as defined by Ferrari and Robertson [9].

**Table 1:** Fitting results of the Raman spectra for various samples

Substrate	Peak (*D*) (cm^−1^)	Peak (*G*) (cm^−1^)	*I_D_*/*I_G_*	*I_2D_*/*I_G_*	FWHM (*G*) (cm^−1^)	FWHM (*2D*) (cm^−1^)
SrTiO_3_	1,372	1,603	0.8	-	70	-
YSZ	1,364	1,609	1.1	-	63	-
SiO_2_	1,352	1,598	1.9	0.4	66	96
Glass	1,352	1,598	1.8	0.3	66	99

Mixed Gaussian and Lorentzian functions are used to fit *D*, *G*, and *2D *peaks. FWHM, full width at half maximum.

The crystalline ordering is worse than that of graphitic carbon grown at the same *T*_G _on a sapphire crystal, where a *2D *peak is easily identified [8]. In the previous study, we observed that the crystal orientations of sapphire substrates did not affect the quality of NCG grown on them and speculated that the lattice constants and the substrate symmetry were not critical parameters in the NCG growth by MBE [8]. Then, we expect similar growth on cubic SrTiO_3 _and YSZ, contrary to what we observe. One possible explanation is that the optimum *T*_G _depends on the material. In fact, the Raman spectra in Figure 1 are similar to those of NCG on sapphire grown at 600°C, far lower than the optimum *T*_G _of 1,100°C [8]. Because of the difference in the sticking coefficient of carbon to the substrate and/or the diffusion constant of carbon on the surface, the optimum growth temperature may depend on the substrate. Further experiments of carbon growth on SrTiO_3 _or YSZ at different temperatures might prove this assumption.

**Figure 2:**
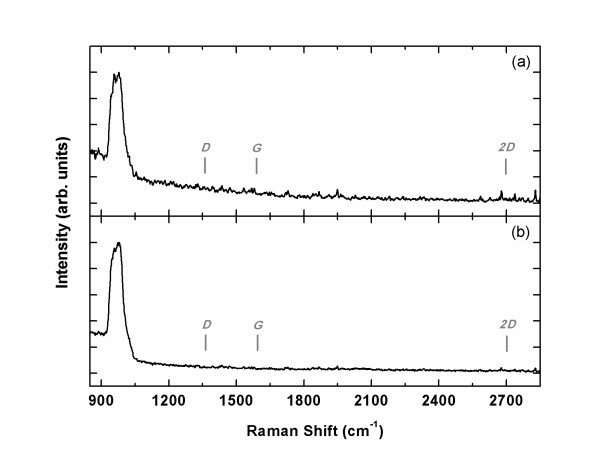
**Raman spectra of carbon films**. The films were grown (**a**) at 900°C on amorphous TiO_2 _and (**b**) at 900°C on amorphous Ta_2_O_5_. No carbon-related peaks are observed. The peak near 1,000 cm^−1 ^is from Si substrate.

Now, we turn to amorphous oxides, which are more relevant to the MOS technology. First, we tested 100-nm-thick TiO_2 _and Ta_2_O_5 _grown on SiO_2_(300 nm)/Si by sputtering. As shown in Figure 2, no sign of graphitic carbon is observed. The only peak near 1,000 cm^−1 ^is the background Raman signal from Si wafer. Usually, this background is removed to highlight the carbon-related peaks, but we leave that in Figure 2 to show the absence of other peaks.

**Figure 3:**
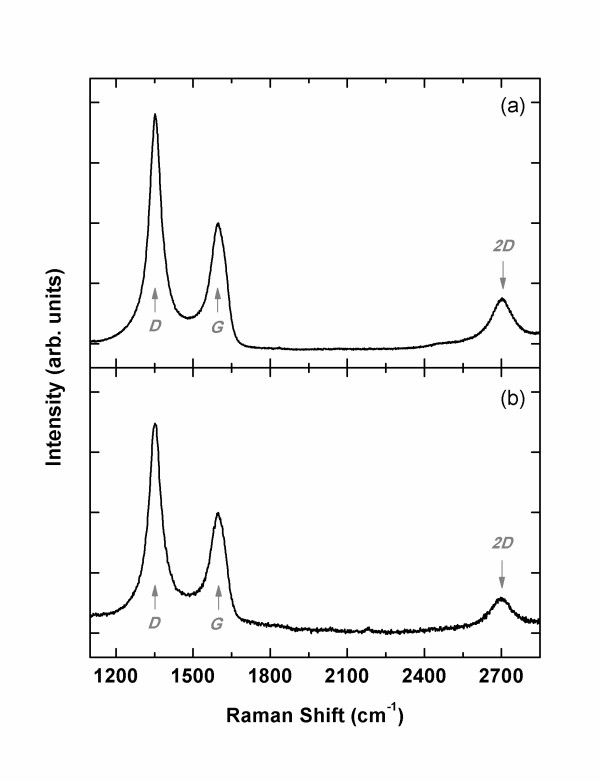
**Raman spectra of carbon films**. The films were grown (**a**) at 950°C on amorphous SiO_2 _and (**b**) at 900°C on Eagle 2000™ glass. In both cases, graphitic carbons of high crystallinity are fabricated.

The situation changes drastically as the substrate is changed to SiO_2_(300 nm) on Si wafer. Figure 3a shows that graphitic carbon of a relatively high degree of crystallinity is formed on SiO_2_. The Raman spectra are similar to the best data from NCG on sapphire [8]: the sharp and large *D *peak and the clear *2D *peak. Notably, the existence of *2D *peak is an important evidence of successful NCG growth on amorphous SiO_2 _[11]. This shows that the crystallinity of the substrate is not essential and explains why the quality of NCG was independent of substrate orientation in the previous study [8]. This surprising result may find interesting applications because we also expect a Dirac-like conduction in NCG [8]. Further optimization along with transport measurement is under progress. Similar results are obtained from Eagle 2000™ glass, a widely used material in active matrix liquid crystal displays (Figure 3b). This glass is known to consist of SiO_2_, B_2_O_3_, Al_2_O_3_, CaO, and Na_2_O. It means that SiO_2 _is not the only amorphous oxide on which graphitic carbon can be fabricated. Considering the variety of oxides, the quality of graphitic carbon can be improved much as the search for suitable substrates is continued.

Now that the carbon films grown on SiO_2 _and glass by MBE are identified as NCGs, it is informative to calculate the crystallite size from Ferrari and Robertson's model applied to stage 2 [9]. According to the model, the average size *L*_a _is related to *I_D_*/*I_G _*as *I_D_*/*I_G _*= *C L*_a_^2^, where *C *= 0.0055 and *L*_a _in Å. From *I_D_*/*I_G _*= 1.8~1.9 (Table 1), we get *L*_a _= 18.1~18.6 Å. In addition, the position of *G *peak at 1,598 cm^−1 ^is in accordance with the identification of NCG of insignificant doping [9].

**Figure 4:**
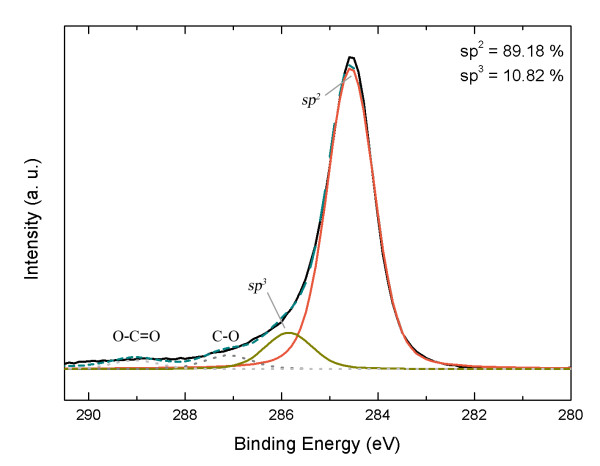
**C1s XPS spectra of graphitic carbon on SiO_2_**. The dashed line is a fit with four Lorentzians. The two strongest peaks (centered at 284.6 eV and 285.8 eV) are assigned to *sp^2 ^*and *sp^3 ^*hybridized carbon atoms, respectively.

In order to clarify the carbon bonding nature, we performed XPS measurements on the graphitic carbon layer on SiO_2_. Figure 4 shows the C1s spectra, which are decomposed into several Lorentzian peaks. Here, we focus on the two strongest peaks centered at 284.6 eV and 285.8 eV. The relative intensity ratios are 89.18% (the peak at 284.6 eV) and 10.82% (the peak at 285.8 eV). In the literature, 284.7 ± 0.2 and 285.6 ± 0.2 eV components are attributed to *sp^2 ^*and *sp^3 ^*hybridization of C-C or C-H bonds, respectively [14]. In combination with the Raman spectra, the XPS results demonstrate that the *sp^2 ^*bonds are dominant in the carbon layer on SiO_2_.

**Figure 5:**
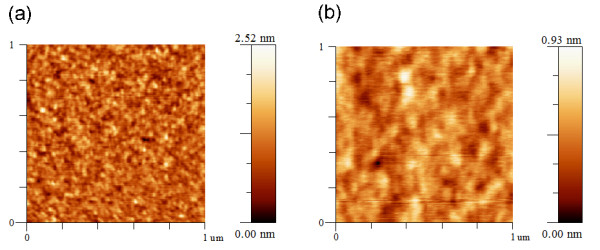
**AFM images of graphitic carbon**. 1 μm × 1 μm AFM images of graphitic carbon on (**a**) SiO_2 _and (**b**) Eagle 2000™ glass. The mean roughness parameters, *R*_a_, from 1 μm × 1 μm scans are (a) 0.224 nm and (b) 0.089 nm, respectively.

Another important result of this work is that the graphitic carbon on amorphous oxide is very flat, which is an important virtue for the integration with other materials. Figure 5 shows the AFM images of graphitic carbon on SiO_2 _and Eagle 2000™ glass. Like the NCG on sapphire, no sign of island growth is observed. The mean roughness parameters, *R*_a_, from 1 μm × 1 μm scans are 0.224 nm (on SiO_2_) and 0.089 nm (on Eagle 2000™ glass). Notably, the *R*_a _of NCG on Eagle 2000™ glass is almost the same as that of the substrate itself which is famous for surface flatness.

## Conclusions

In summary, we have grown graphitic carbon on crystalline and amorphous oxides by using carbon MBE. Notably, the graphitic carbons on amorphous SiO_2 _and on glass show a relatively high degree of graphitization, evidenced by well-developed *D*, *G*, and *2D *Raman peaks. The C1s spectra from XPS measurements confirm the dominance of *sp^2 ^*carbon bonding. In addition, the surfaces are almost as flat as the substrates, which may play an important role in the integration with the existing technology.

## Abbreviations

AFM: atomic force microscopy; CVD: chemical vapor deposition; MOS: metal-oxide semiconductor; MBE: molecular beam epitaxy; NCG: nanocrystalline graphite; XPS: X-ray photoelectron spectroscopy; YSZ: yttria-stabilized zirconia.

## Competing interests

The authors declare that they have no competing interests.

## Authors' contributions

SKJ carried out the carbon molecular beam epitaxy experiments and X-ray photoelectron spectroscopy. DSY participated in the carbon molecular beam epitaxy experiments. JHL carried out the atomic force microscopy measurements. CK and SY characterized the thin films by Raman spectroscopy. SHC designed the experiments and wrote the manuscript. All authors read and approved the final manuscript.
